# Evaluation of multidetector CT Hounsfield unit measurements as a predictor of efficacy and complications in percutaneous vertebroplasty for osteoporotic vertebral compression fractures

**DOI:** 10.3389/fmed.2023.1333679

**Published:** 2023-11-29

**Authors:** Dimo Yankov, Assen Bussarsky, Vasil Karakostov, Alexander Sirakov, Dilyan Ferdinandov

**Affiliations:** ^1^Clinic of Neurosurgery, St. Ivan Rilski University Hospital, Sofia, Bulgaria; ^2^Department of Neurosurgery, Faculty of Medicine, Medical University of Sofia, Sofia, Bulgaria; ^3^Department of Radiology, St. Ivan Rilski University Hospital, Sofia, Bulgaria

**Keywords:** percutaneous vertebroplasty, Hounsfield units, cement leakage, VAS, ODI

## Abstract

**Introduction:**

More than 30 years after the initial experience of Galibert and Deramond with percutaneous vertebroplasty, the procedure has gone through countless refinements and clinical evaluations. Predictors for the success and failure of the procedure in the literature vary and are focused on the duration of complaints, type of fracture, presence of edema on MRI scans, etc. We propose using a quantitative method based on a standard CT examination of the thoracic or lumbar spine to assess the risks and potential success of performing vertebroplasty.

**Materials and methods:**

This is a single-center prospective observational study on 139 patients treated with percutaneous vertebroplasty (pVPL) for a single symptomatic osteoporotic vertebral compression fracture (OVCF). We measured the levels of disability and pain preoperatively and again at the 3-, 6- and 12-month marks using the standardized VAS and ODI questionnaires. Every patient in the study was evaluated with postoperative multidetector CT (MDCT) to determine the presence, extent, and localization of vertebral cement leakage and to measure the adjacent vertebrae’s minimal and mean density in Hounsfield units (HU_min_ and HU_mean_, respectively).

**Results:**

We determined that a slight (*r* = −0.201) but statistically significant (*p* = 0.018) correlation existed between HU measurements taken from radiologically intact adjacent vertebrae and the procedure’s effect concerning the pain levels at the 3-month follow-up. This correlation failed to reach statistical significance at 12 months (*p* = 0.072). We found no statistically significant relationship between low vertebral cancellous bone density and cement leakage on postoperative scans (*p* = 0.6 for HU_min_ and *p* = 0.74 for HU_mean_).

**Conclusion:**

We have moderately strong data that show a negative correlation between the mean values of vertebral cancellous bone density in patients with OVCF and the effect of pVPL in reducing pain. Lower bone densities, measured this way, showed no increased risk of cement leakage.

## Introduction

Percutaneous vertebroplasty (pVPL) was initially applied in the treatment of symptomatic spinal hemangioma (1) and subsequently used in various other pathologies, including spinal osteolytic neoplasms (2) and simple osteoporotic vertebral compression fractures (OVCFs) (3). The procedure has been through the gauntlet of the RCT multiple times with varying results and recommendations (4–7). To date, most authors agree that pVPL is a viable alternative after conservative management has failed to produce pain control (8–14).

Like most other invasive medical procedures, percutaneous vertebroplasty is not without complications. A systematic review conducted by Hulme et al. (15) suggests that these should be separated into two categories:

1. Procedural – bone fractures, nerve, and pressure injuries due to improper positioning of the patient on the operating table; intervention site infection; cardiopulmonary suppression due to intraoperative use of opioids; iatrogenic injury of neural and vascular structures due to suboptimal placement of working cannulas, and others.
2. Complications secondary to cement leakage outside the vertebral body include pulmonary artery embolization, spinal canal occlusion, thermal and compression injuries due to exothermal polymerization, and hardening of the compound near neural structures.

Since the first category is not exclusive to pVPL and these complications can be observed in any surgery performed in the prone position (16), we will evaluate the risk factors affecting cement leakage, a complication that is bespoke to vertebral augmentation procedures.

Even though cement leakage following pVPL is frequent, actual adverse clinical events are very few and quite rare (17). The current literature on the evaluation of risk factors for cement leakage is focused on fracture severity, bone cement dispersion types, puncture approach, presence of cortical surface disruption, and others (18). A recent study by Jun Liu et al. suggests that bone mineral density, measured with dual-energy X-ray absorptiometry (DXA), can predict the dispersion pattern of cement during the procedure (19). Another study has shown a causal link between low bone mineral density and a higher incidence of cement leakage during pVPL (20).

Multiple studies have shown a strong relationship between bone mineral density values from DXA and HU measurements taken from vertebrae, thorax, pelvis, cranium and other bones (21–24). Additionally, quantitative computed tomography (qCT) has been recognized as an alternative to DXA in diagnosing osteoporosis since the latter half of the 1970s (25). Building further on these well-established dependencies, we aim to investigate any existing relationship between HU measurements obtained via MDCT and the therapeutic effect of pVPL and the incidence and complications stemming from cement leakage.

## Materials and methods

### Patient selection and data collection

The present study is a single-center prospective observational study on 139 patients. The inclusion criteria for the study were as follows: single symptomatic OVCF determined by the presence of bone marrow edema on STIR MRI images or by bone scintigraphy when MRI was contraindicated. VAS pain score ≥ 5 after optimal conservative management, including physiotherapy; duration of symptoms no more than six months; spontaneously occurring fractures due to fragility or minimal traumatic etiology (e.g., during otherwise physiological physical exertion, improper movements, lifting a heavy object, minor daily injuries that would not lead to a fracture in the absence of osteoporosis). Exclusion criteria: evidence of malignancy in any of the scanned vertebrae; dementia or inability to understand or complete the needed forms; history of significant back pain before the incidence of OVCF; history of rheumatological disease affecting the spine other than osteoporosis; lack of radiologically intact adjacent vertebrae on the postoperative CT scans.

Between January 2018 and January 2022, a total of 1,025 vertebroplasty procedures were performed for the treatment of various pathologies in the Clinic of Neurosurgery at St. Ivan Rilski University Hospital, Sofia, Bulgaria. Of those, 157 patients fit the inclusion criteria. Participants were required to consent to postoperative CT imaging. N = 18 declined participation, most of them citing unnecessary ionizing radiation exposure as the main reason. The remaining 139 patients who underwent pVPL for a single-level OVCF were enrolled in the study.

The patient data recorded were age, sex, level of the fractured vertebra, degree of vertebral fracture, pain, and disability levels, measured by the standardized VAS and ODI questionnaires, (26, 27) – before surgery and at 3-month intervals during the 12-month follow-up. Additionally direct pain control was assessed at 24 h after the procedure. A postoperative, noncontrast CT scan of the affected spinal segment was taken to assess the presence and location of cement leakage and to measure HU_min_ and HU_mean_ from radiologically intact adjacent vertebrae. The severity of the fractures was determined via intraprocedural fluoroscopy using the semiquantitative grading method described by Genant et al.: Grade 1: mild, ≤25% loss of body height; Grade 2: moderate, 25–40% loss; Grade 3: severe, >40% loss (28). These were then further subcategorized to account for the presence of an intravertebral cleft sign, a.k.a. Kümmell disease (29).

All 139 patients received a thoracic and lumbar spine X-rays at the end of the follow-up to assess for the presence new OVCFs. If findings were inconclusive, an MRI of the suspected segment was also performed.

### MDCT examination

All postoperative scans were performed on a 16-slice MDCT scanner (GE BrightSpeed) at the St Ivan Rilski University Hospital. The CT parameters for the study were as follows: peak potential 120 kVp, slice thickness of 1.25 mm, and 2–3 mm increments. A radiologist and two neurosurgeons independently performed the image evaluation. Complete unanimity was required to classify a radiological artifact as a cement leakage since it could be minuscule in volume.

**Figure 1:**
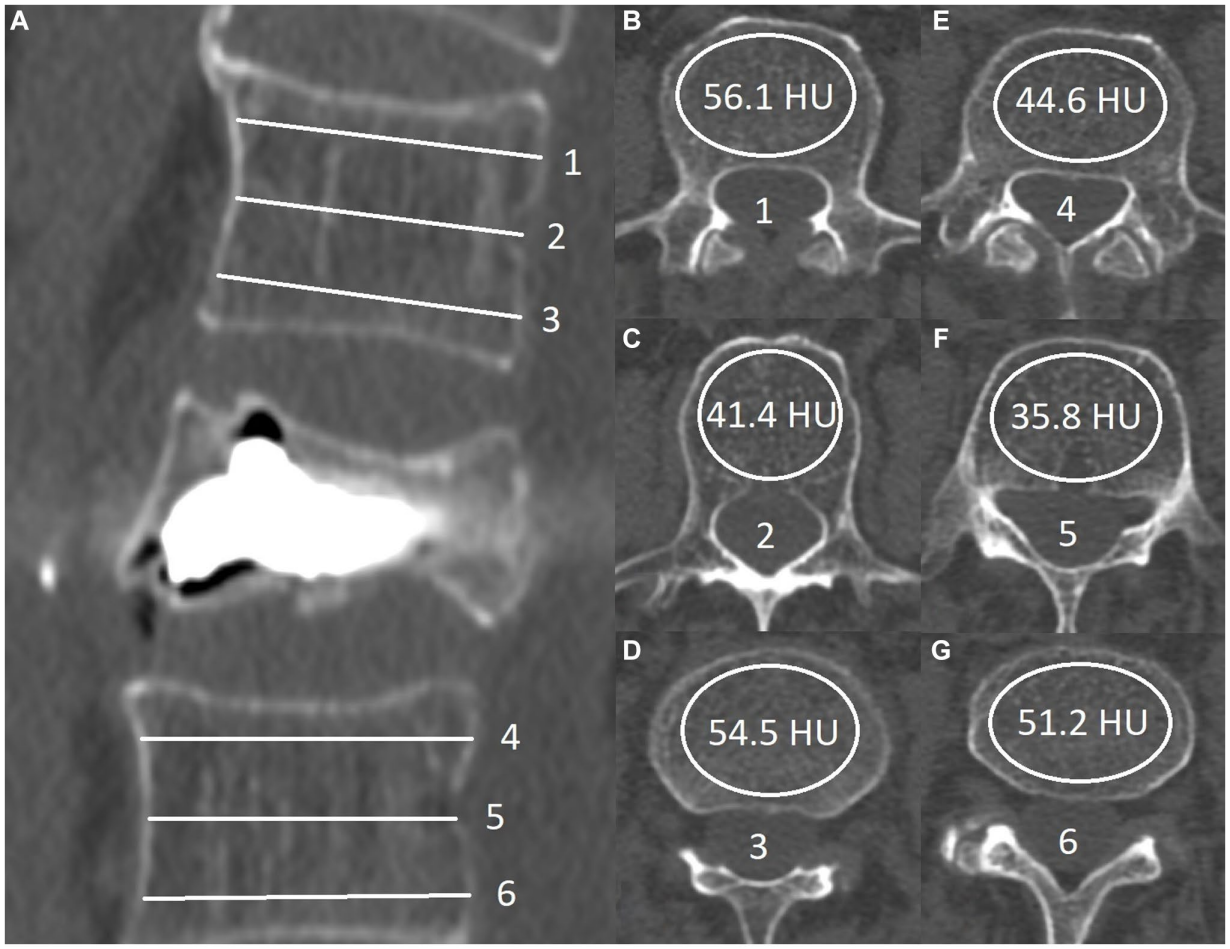
Sagittal view of postprocedural computed tomography: **(A)** Six transverse lines represent the level of the ROI measurement **(B-G)**. HU_mean_ values are calculated by the formula (56.1 + 41.4 + 54.5 + 44.6 + 35.8 + 51.2)/6 = 47.3. The HU_min_ value in this patient was 35.8 **(F)**.

The study’s main variables, HU_min_ and HU_mean_, were measured using standard DICOM viewing software to draw oval regions of interest (ROIs) inside the body of the two closest, radiologically intact, adjacent vertebrae, excluding their cortical surfaces. HU_mean_ was estimated arithmetically from the value taken using six axial cut slices, three from each vertebra. A technique similar to the one described by Schreiber et al. (22). In the original paper, the authors observe a statistically significant correlation in the values taken from a single intact vertebra and BMD scores obtained via DXA. However, we propose that modifying the technique by using 2 immediately adjacent vertebrae (one above and one below) would yield measurements that more closely represent the density within the fractured vertebrae before vertebral body collapse and secondary compaction of the cancellous structure had occurred (Figure 1).

The minimal observed HU (HU_min_) values were also used in the subsequent statistical analysis; these were often lower by more than 50% from the observed HU_mean_. The HU_min_ quoted here is the lowest of all six measures and not the minimal value of radiodensity inside each separate ROI. In instances where subchondral osteosclerosis was present in the adjacent vertebrae, we took measurements from the two closest vertebrae that appeared radiologically intact. This approach eliminates the artificially heightened HU_mean_ results that these radiologically denser lesions would cause.

**Figure 2:**
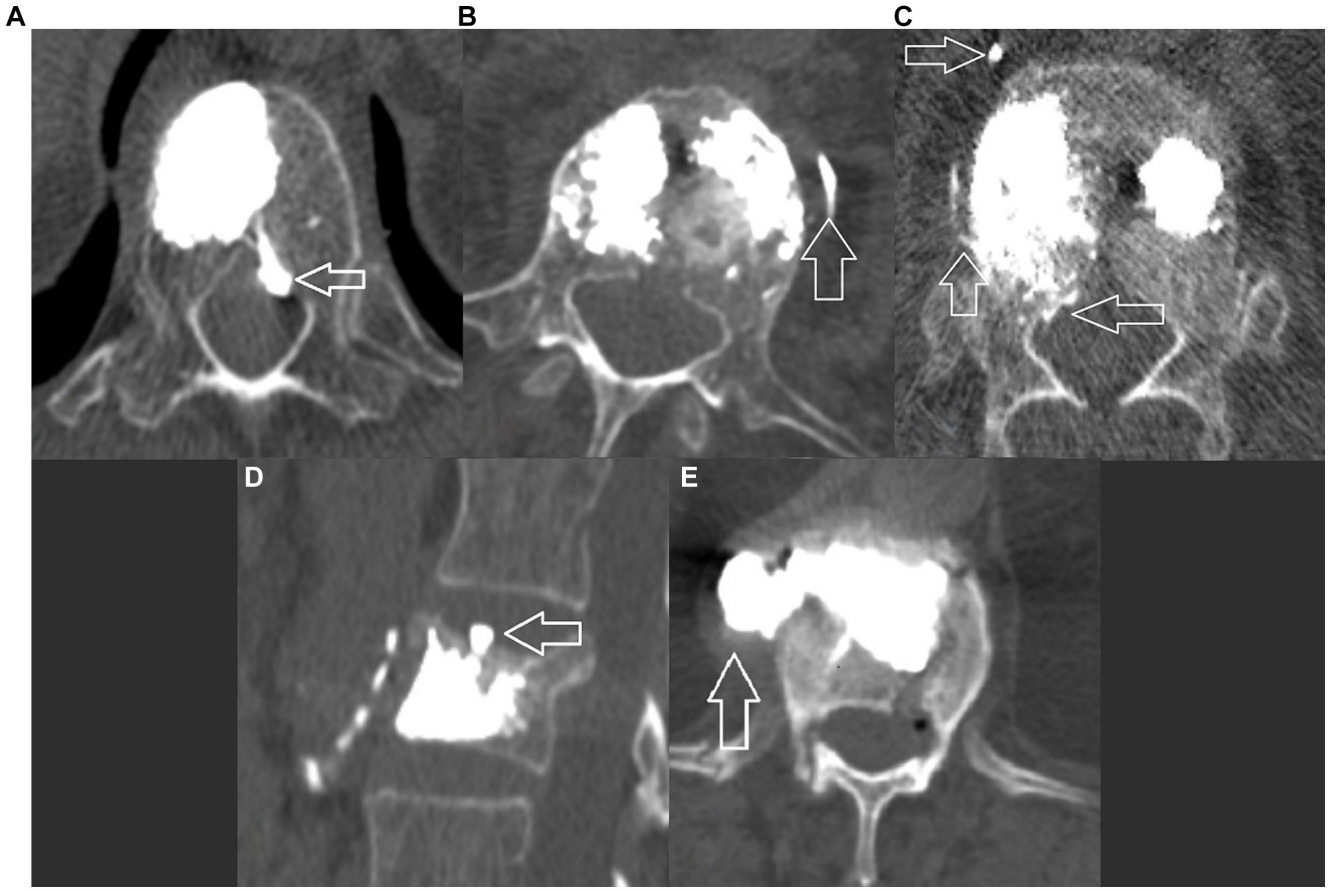
Postoperative CT, axial view showing different categories of leakage: **(A)** Type A – inside the spinal canal through the basivertebral vein; **(B)** Type B – inside the segmental veinous outflow; **(C)** Type C – inside the spinal canal and the segmental veinous outflow; **(D)** Type D inside the intervertebral disk; and **(E)** Type E – in the paravertebral space, leak occurs through a cortical defect.

Any presence of vertebral cement outside of the cortical contour of the target vertebra on the CT scan was noted and further subclassified into five categories (Figures 2A–E).

Percutaneous vertebroplasty was performed using local anesthesia under fluoroscopic guidance via a bipedicular approach. A standard high-viscosity cement was used in all procedures. Cement injection is carried out until optimal vertebral body fill is observed (30) or there is fluoroscopic evidence of extravertebral cement leakage. Note that isolated leakage into the intervertebral disk was not considered grounds to terminate the procedure, as current evidence suggests that this type of cement leak has no negative predictive value for the overall success of the procedure and does not contribute to any clinically significant complications (31). The volume of cement applied was recorded at the end of the procedure.

### Statistical analysis

Univariate analysis was used to determine the relationship between HU_min_ and HU_mean_ values and the development of specific grade fractures, pain (VAS), and disability (ODI) levels. Furthermore, logistic regression analysis was used to determine the relationship between these variables and bone cement leakage on postoperative CT scans. These tests were then subcategorized for each distinctive type of cement leak. The following risk factors were evaluated: severity of fracture and the presence of Kümmell disease. The statistical analyses were conducted using SPSS Version 19 (IBM, NY, United States). *p*-values at or below 0.05 were regarded as significant.

## Results

Baseline VAS pain and ODI disability levels were established for all 139 participants. The mean duration of complaints was 11 weeks (±9; 2–26). In addition to receiving a postoperative CT scan on the following day, VAS levels were also reassessed to determine the immediate effect of the procedure. We performed neurological examination and re-evaluation of VAS and ODI at 3-, 6- and 12-month time points. The demographics, baseline scores and immediate postoperative results of all patients enrolled in the study (N = 139) are presented in Table 1. The HU_min_ and HU_mean_ values measured for each age group are summarized in Table 2.

**Table 1:** Demographic characteristics of all patients, baseline pain (VAS) and disability (ODI), immediate postoperative pain (VAS), and type of fracture (mean ± SD).

	All patients, *N* = 139	Female, *N* = 104	Male, *N* = 35	Presence of IVC sign, *N* = 35	Fracture grade		
					Grade I, *N* = 27	Grade II, *N* = 60	Grade III, *N* = 52
Age	70.8 ± 9.6	70.2 ± 10.1	72.7 ± 7.8	72.7 ± 10.6	67.4 ± 8.9	71.9 ± 9.4	71.3 ± 10.0
VAS pre	7.7 ± 1.6	7.6 ± 1.6	7.9 ± 1.5	8.9 ± 1.2	6.7 ± 1.4	72.3 ± 1.5	8.6 ± 1.2
VAS post	2.9 ± 1.6	2.9 ± 1.6	2.8 ± 1.4	2.6 ± 1.3	2.9 ± 2.1	2.8 ± 1.6	3.1 ± 1.2
ODI(%) pre	52.9 ± 12.3	52.7 ± 12.5	53.4 ± 11.8	60.5 ± 10.1	45.3 ± 8.1	50.5 ± 12.0	59.6 ± 11.3
Cement leak, *N* (%)	54 (38.9%)	40 (38.5%)	14 (40.0%)	8 (22.9%)	10 (37.0%)	27 (45.0%)	17 (32.7%)
Type A	3	2	1	0	1	3	0
Type B	12	9	3	0	2	5	5
Type C	8	6	2	0	4	4	0
Type D	27	20	7	6	4	13	10
Type E	4	3	1	2	0	2	2

**Table 2:** Hounsfield unit (HU) measurements by age group.

			HU_min_ values		HU_mean_ values	
Age group	Female, N = 104	Male, N = 35	Female	Male	Female	Male
50–59	20 (19.2%)	2 (5.7%)	59.2	61.3	99.1	117.7
60–69	29 (27.9%)	9 (25.7%)	38.4	42.4	71.6	95.8
70–79	33 (31.7%)	17 (48.6%)	5.1	39.8	43.3	62.3
≥80	22 (21.2%)	7 (20.0%)	−24.7	19.5	34.2	54.2

Thirty-seven of the 139 patients did not complete the predetermined 12-month follow-up: N = 10 completed the 3-month follow-up but were later lost without contact (N = 7) or were reported as deceased (N = 3); N = 17 completed the 6-month follow-up, subsequently N = 12 were lost without contact, two were diagnosed with a primary malignancy, two were reported as deceased, and one was diagnosed with fibromyalgia. 10 patients had evidence of a new OVCF on the X-ray reevaluation taken at the 12-month mark. They were excluded from the statistical analysis at the 12-month mark since they no longer fit the criteria for a single symptomatic OVCF. The demographic data, CT data, and direct postoperative results for the patients who did not complete the 12-month follow-up (N = 37) were used in the statistical analysis wherever appropriate, e.g., immediate postoperative results for pain control and cement leakage.

**Figure 3:**
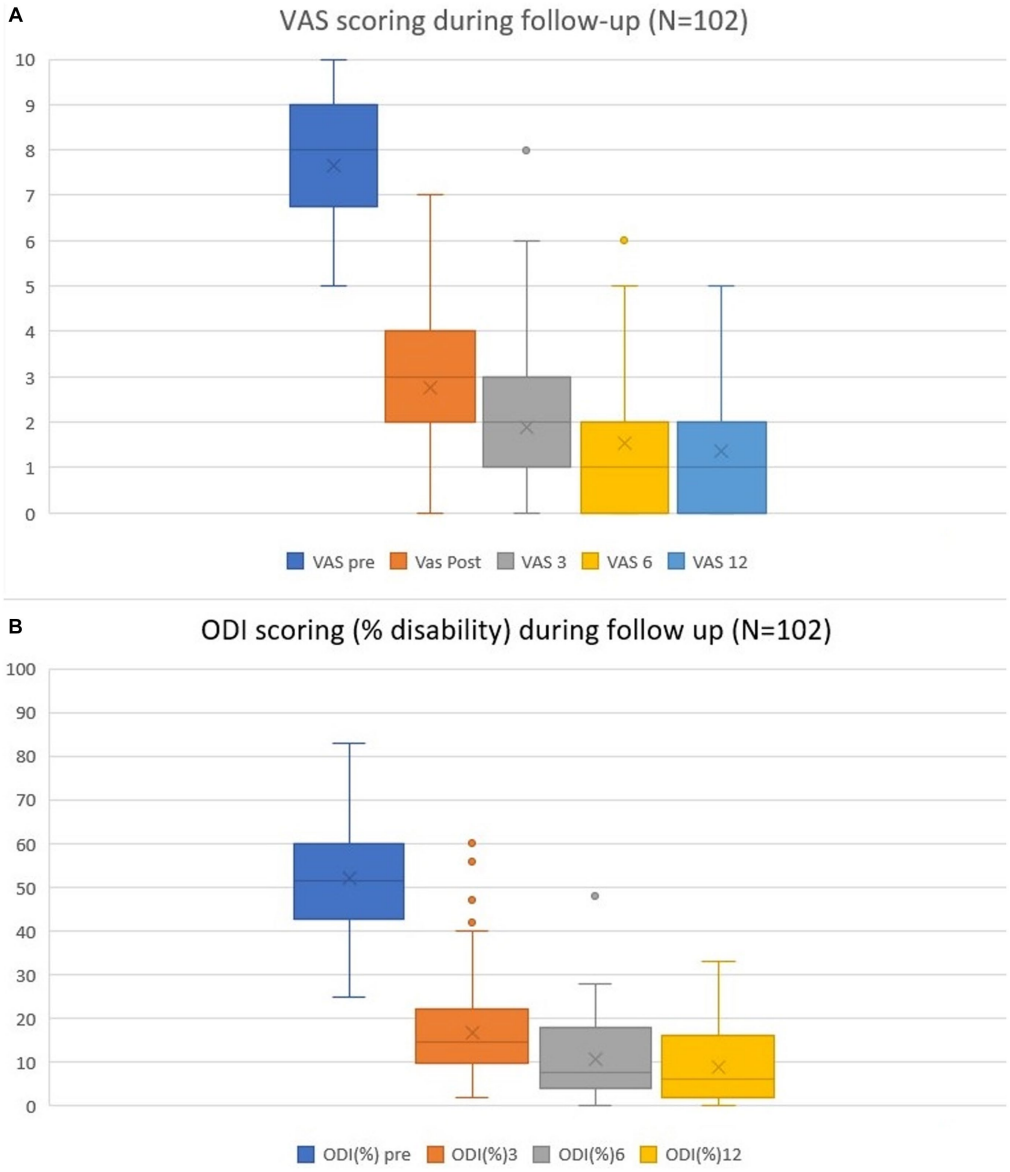
Pain VAS **(A)** and disability ODI **(B)** scores preoperatively and during follow-up at the 1-day, 3-, 6- and 12-month time points. Mean values, ranges, and standard deviations are given.

None of the patients (N = 139) exhibited any intra- or postoperative adverse events. Everyone who went through the 12-month follow-up (N = 102) revealed a significant reduction in overall VAS and ODI scores (Figure 3). The mean preoperative VAS score was 7.7 (±1.5; range 5–10). On the first postoperative day, this score was 2.8 (±1.5; 0–7). The 3- and 6-month follow-up results became more linear, with mean scores of 1.9 (±1.6; 0–8) and 1.5 (±1.5; 0–6), respectively. The mean total reduction (∆VAS) for the follow-up was 6.4 ± 1.7. Disability scoring followed a similar trend: the preoperative mean scores were 52 ± 12.5, 17 ± 10.7% at 3 months, 11% ± 9.2% at 6 months, and 9.0% ± 10.7% at 12 months. The overall mean reduction in ∆ODI was 42.0% ± 13.3%.

We found a correlation near statistical significance (*p* = 0.056) between the mean values and the preoperative VAS scores (*r* = −0.190). This would suggest that a decrease in overall density is a predictor for higher VAS scores before the procedure. This correlation was significant when comparing HU mean and VAS scores at the 3-month follow-up (*p* = 0.018; *r* = −0,201). However, these correlations failed to reach statistical significance when measuring preoperative disability (*p* = 0.223) or the effect of the procedure in reducing pain (∆VAS) and disability (∆ODI) scores at the end of 12 months (*p* = 0.516 and *p* = 0.968, respectively) (Table 3).

**Table 3:** Univariate analysis for all patients who completed the 12-month follow-up (N = 102).

		Pearson Correlation test		Spearman’s rho		Kendall’s tau B	
		HUmin	HUmean	HUmin	HUmean	HUmin	HUmean
VAS preoperative	r	−0.141	−0.190	−0.150	−0.190	−0.110	−0.137
	*p* - value	0.158	0.056	0.133	0.056	0.130	0.058
VAS on postoperative day one	r	0.040	−0.007	0.031	−0.015	0.025	−0.012
	*p* - value	0.688	0.943	0.759	0.884	0.732	0.871
VAS at 3-month follow-up	r	−0.152	−0.201	−0.143	−0.185	−0.101	−0.132
	*p* - value	0.075	0.018	0.095	0.030	0.104	0.034
VAS at 12-month follow-up	r	−0.174	−0.179	−0.166	−0.186	−0.127	−0.139
	*p* - value	0.081	0.072	0.095	0.061	0.088	0.062
Total reduction in pain – ∆VAS	r	−0.026	−0.065	−0.068	−0.092	−0.050	−0.075
	*p* - value	0.797	0.516	0.495	0.357	0.488	0.296
ODI(%) preoperative	r	−0.096	−0.122	−0.110	−0.135	−0.077	−0.093
	*p* - value	0.338	0.223	0.271	0.175	0.259	0.175
Total reduction in disability – ∆ODI(%) at 12-month follow-up	r	0.041	0.004	0.011	−0.030	0.001	−0.026
	*p* - value	0.682	0.968	0.914	0.761	0.988	0.705

Fifty-four patients had evidence of cement leakage on postoperative CT. These were subclassified as type A (*N* = 3) - through the basivertebral vein inside the spinal canal; type B (*N* = 12) in the segmental venous outflows; type C (*N* = 8) as a combination of A and B; type D (*N* = 27) inside the intervertebral disk; and type E (*N* = 4) – paravertebral through a defect in the cortical surface of the vertebrae.

We observed that the presence of the intravertebral vacuum clef (IVC sign, a.k.a. Kümmell’s disease) was a protective factor against cement leakage (*p* = 0.048; x^2^ = 11,165) and further protective against subtypes A-C, since all patients in the study with evidence of clefts on preoperative images only exhibited D- and E-type cement leakage. Grade 3 fracture severity was determined to be a protective factor against type A leakage. None of these patients (N = 52) exhibited PMMA leakage through the basivertebral vein toward the spinal canal. The volume of injected bone cement was a risk factor for these complications overall (*p* < 0.05) but was not significant (*p* = 0.215) in the presence of the IVC sign. The latter is only valid with volumes of PMMA ≤12 cm^3^ since commercially available systems used in this trial are limited to this amount.

Cancellous bone density, measured in HU_min_ and HU_mean_, showed no correlation to the incidence of cement leakage overall (*p* = 0.233, *x*^2^ = 6.842, and *p* = 0.415, *x*^2^ = 5.005, respectively) or to the likelihood of developing each of the subtypes (Table 4).

**Table 4:** Multivariate logistic regression analysis for the development of each fracture subtype.

Cement leak category		В	Standard error	Wald	*p*	Exp(B)	95% CI	
							Lower bound	Upper bound
A	Intercept	−5.149	1.693	9.254	0.002			
	HU_min_	0.042	0.066	0.401	0.526	1.043	0.916	1.187
	HU_mean_	−0.004	0.062	0.004	0.948	0.996	0.881	1.126
B	Intercept	−2.578	0.945	7.451	0.006			
	HU_min_	0.072	0.046	2.440	0.118	1.075	0.982	1.176
	HU_mean_	−0.047	0.045	1.107	0.293	0.954	0.874	1.041
C	Intercept	−4.130	1.154	12.816	0.000			
	HU_min_	0.017	0.047	0.138	0.711	1.017	0.929	1.115
	HU_mean_	0.012	0.044	0.078	0.780	1.012	0.929	1.103
D	Intercept	−2.115	0.616	11.773	0.001			
	HU_min_	−0.026	0.028	0.875	0.349	0.974	0.922	1.029
	HU_mean_	0.035	0.026	1.774	0.183	1.036	0.984	1.091
E	Intercept	−3.295	1.435	5.271	0.022			
	HU_min_	0.096	0.072	1.773	0.183	1.100	0.956	1.267
	HU_mean_	−0.069	0.070	0.961	0.327	0.933	0.813	1.071

## Discussion

To our knowledge, this is the first study that evaluates the correlation of quantitative Hounsfield unit measurements to postoperative results from percutaneous vertebroplasty and the risk of cement leakage. Previously reported risk factors for this complication as well as suboptimal pain control from the procedure include patient age, duration of complaints, the volume of applied bone cement, cement dispersion patterns, the severity of the fracture, bone mineral density measurements by DXA, and others (18, 32–34).

In the present study, bone cement leakage was observed in 43 cases (~27%) via intraoperative fluoroscopy. Postoperative CT showed evidence of extravertebral PMMA leakage in another 11 patients for a total of 54 (~40%) cases of PMMA leakage. The difference in reporting by intraoperative fluoroscopy and postoperative CT is well established. This study falls within the average incidence of cement leakage (31, 33). However, there is ambiguity in the literature and no threshold is determined as to what should be considered noteworthy PMMA leak. Therefore, these results should be observed, considering that in some cases this volume is minuscule. Additionally, we must note that postoperative CT was performed for the region of interest, either the thoracic or lumbar spine, and in most cases was not representative of the distal venous outflows and pulmonary arteries. We cannot definitively conclude that distal embolic complications were not present away from the site of intervention/scanning.

We observed that the presence of an IVC sign was a protective factor against all forms of transvenous cement leakage (Figures 2A–C). As described by Tome-Bermejo et al., these clefts are secondary to avascular necrosis of the bone, resulting in a low pressure-low density zone, similar to that created by inflating the balloon during percutaneous kyphoplasty. The PMMA fill pressure is considerably lower than that needed in intact and/or secondarily compacted cancellous bone. The authors hypothesize that the collapse of the normal trabecular structure inside the body destroys any venous channels or interrupts their connection to the basivertebral and segmental veins (34).

Lower densities measured by CT did not appear to correlate to a higher incidence of PMMA cement leakage. This finding contradicts previous reports that used DXA scans to measure bone density (20). While we cannot be certain why such a discrepancy exists, most other authors agree that the severity of the fracture, presence of IVCs and disruption of the cortical wall of the vertebrae are the major predictive factors for cement leakage, and bone mineral density does not play a major role (18, 32, 34).

These measurements did not correlate significantly with the overall reduction in pain and disability scores. However, there was a slight (*r* = −0,201) statistically significant (*p* = 0.018) correlation between the HU_mean_ measurements and VAS score at the 3-month follow-up, i.e., patients with lower density scores could be at a higher risk for inadequate pain control in the short term. This correlation is not strong enough to constitute a clinical guideline or predictive model.

The current study has a new fracture rate of approximately 7%. These patients were excluded from the statistical analysis overall, apart from direct postoperative pain control and CT-defined cement leakage. The relatively small number of patients in the current study (N = 139) and the low rate of new OVCFs (*N* = 10) prohibit us from drawing any statistically significant conclusions on the correlation between HU_min_ and HU_mean_ values and new fractures. However, there is ample evidence in the literature linking low BMD and patient age to the incidence of new fractures. The meta-analysis conducted by Hui Zhang et al., published in 2017, decisively dissociated pVPL as a risk factor in the occurrence of new OVCFs. The authors conclude that persisting low BMD T scores <−3 SD and patient age > 80 constitute the major risk factors, while BMI, tobacco smoking, low serum vitamin D and others are secondary and do not contribute as much to the overall risk (35).

### Limitations of the study

This prospective study’s relatively small number of patients limits its significance. Thus, we cannot propose a guideline based on these findings. Further investigation within a larger demographic could influence the strength of these statistical correlations. Additionally, this single-center study is susceptible to observational bias, and using semiquantitative methods to subcategorize fracture types presents observer bias. The tools used to evaluate patient well-being and procedural success (VAS and ODI) are subjective and are prone to reporting biases.

## Conclusion

Our findings suggest a correlation between low bone density measurements and poorer results after percutaneous vertebroplasty in the first three months after the procedure. This correlation is not present at one year. The existence of an intravertebral vacuum cleft sign is a protective factor against cement leakage overall and transvenous cement embolization. Lower HU_min_ and HU_mean_ values did not contribute to a higher incidence of cement leaks. These metrics remained statistically irrelevant to the overall disability of the patients on presentation or at any point during the 12-month follow-up.

## Data availability statement

The original contributions presented in the study are included in the article/supplementary material, further inquiries can be directed to the corresponding author.

## Ethics statement

The studies involving humans were approved by Institutional Ethical Review at St. Ivan Rilski University Hospital, Sofia, Bulgaria. The studies were conducted in accordance with the local legislation and institutional requirements. The participants provided their written informed consent to participate in this study.

## Author contributions

DY: Writing – review & editing, Conceptualization, Formal analysis, Investigation, Methodology, Visualization, Writing – original draft. AB: Methodology, Project administration, Supervision, Writing – review & editing. VK: Project administration, Resources, Supervision, Writing – original draft. AS: Data curation, Formal analysis, Software, Visualization, Writing – original draft. DF: Investigation, Project administration, Resources, Supervision, Visualization, Writing – review & editing.

## References

[1] Galibert P Deramond H Rosat P Le Gars D. Preliminary note on the treatment of vertebral angioma by percutaneous acrylic vertebroplasty. Neurochirurgie. (1987) 33:166–8. 3600949

[2] Manfrè L Guarnieri G Muto M. Vertebroplasty and spinal tumors In: Muto M, editor. Interventional neuroradiology of the spine: Clinical features, diagnosis and therapy [internet]. Milano: Springer Milan (2013). 131–61.

[3] Jensen ME Evans AJ Mathis JM Kallmes DF Cloft HJ Dion JE. Percutaneous polymethylmethacrylate vertebroplasty in the treatment of osteoporotic vertebral body compression fractures: technical aspects. AJNR Am J Neuroradiol. (1997) 18:1897–904. PMID: 9403451 PMC8337380

[4] Buchbinder R Busija L. Why we should stop performing vertebroplasties for osteoporotic spinal fractures. Intern Med J. (2019) 49:1367–71. doi: 10.1111/imj.14628, PMID: 31713338

[5] Buchbinder R Osborne RH Ebeling PR Wark JD Mitchell P Wriedt C. A randomized trial of vertebroplasty for painful osteoporotic vertebral fractures. N Engl J Med. (2009) 361:557–68. doi: 10.1056/NEJMoa0900429 19657121

[6] Kallmes DF Comstock BA Heagerty PJ Turner JA Wilson DJ Diamond TH. A randomized trial of vertebroplasty for osteoporotic spinal fractures. N Engl J Med. (2009) 361:569–79. doi: 10.1056/NEJMoa0900563, PMID: 19657122 PMC2930487

[7] Firanescu CE de Vries J Lodder P Venmans A Schoemaker MC Smeet AJ. Vertebroplasty versus sham procedure for painful acute osteoporotic vertebral compression fractures (VERTOS IV): randomised sham controlled clinical trial. BMJ. (2018) 361:k1551. doi: 10.1136/bmj.k1551, PMID: 29743284 PMC5941218

[8] Clark WC Coscia M Acker JD Wainscott K Robertson JT. Infection-related spontaneous atlantoaxial dislocation in an adult. Case report J Neurosurg. (1988) 69:455–8. doi: 10.3171/jns.1988.69.3.0455, PMID: 3042920

[9] Klazen CAH Lohle PNM de Vries J Jansen FH Tielbeek AV Blonk MC. Vertebroplasty versus conservative treatment in acute osteoporotic vertebral compression fractures (Vertos II): an open-label randomised trial. Lancet. (2010) 376:1085–92. doi: 10.1016/S0140-6736(10)60954-3 20701962

[10] Chen D An ZQ Song S Tang JF Qin H. Percutaneous vertebroplasty compared with conservative treatment in patients with chronic painful osteoporotic spinal fractures. J Clin Neurosci. (2014) 21:473–7. doi: 10.1016/j.jocn.2013.05.017 24315046

[11] Blasco J Martinez-Ferrer A Macho J San Roman L Pomés J Carrasco J. Effect of vertebroplasty on pain relief, quality of life, and the incidence of new vertebral fractures: a 12-month randomized follow-up, controlled trial. J Bone Miner Res. (2012) 27:1159–66. doi: 10.1002/jbmr.1564, PMID: 22513649

[12] Farrokhi MR Alibai E Maghami Z. Randomized controlled trial of percutaneous vertebroplasty versus optimal medical management for the relief of pain and disability in acute osteoporotic vertebral compression fractures. J Neurosurg Spine. (2011) 14:561–9. doi: 10.3171/2010.12.SPINE10286, PMID: 21375382

[13] Rousing R Hansen KL Andersen MO Jespersen SM Thomsen K Lauritsen JM. Twelve-months follow-up in forty-nine patients with acute/semiacute osteoporotic vertebral fractures treated conservatively or with percutaneous vertebroplasty: a clinical randomized study. Spine. (2010) 35:478–82. doi: 10.1097/BRS.0b013e3181b71bd1 20190623

[14] Voormolen MHJ Mali WPTM Lohle PNM Fransen H Lampmann LEH van der Graaf Y. Percutaneous vertebroplasty compared with optimal pain medication treatment: short-term clinical outcome of patients with subacute or chronic painful osteoporotic vertebral compression fractures. The VERTOS study. AJNR Am J Neuroradiol. (2007) 28:555–60. PMID: 17353335 PMC7977842

[15] Hulme PA Krebs J Ferguson SJ Berlemann U. Vertebroplasty and Kyphoplasty: a systematic review of 69 clinical studies. Spine. (2006) 31:1983–2001. doi: 10.1097/01.brs.0000229254.89952.6b, PMID: 16924218

[16] Kwee MM Ho YH Rozen WM. The prone position during surgery and its complications: a systematic review and evidence-based guidelines. Int Surg. (2015) 100:292–303. doi: 10.9738/INTSURG-D-13-00256.1, PMID: 25692433 PMC4337445

[17] Eck J Nachtigall D Humphreys S Hodges S. Comparison of vertebroplasty and balloon kyphoplasty for treatment of vertebral compression fractures: a meta-analysis of the literature. Orthopedics and Physical Rehabilitation Publications. (2008) 8:488–97. doi: 10.1016/j.spinee.2007.04.004 17588820

[18] Zhang K She J Zhu Y Wang W Li E Ma D. Risk factors of postoperative bone cement leakage on osteoporotic vertebral compression fracture: a retrospective study. J Orthop Surg Res. (2021) 16:183. doi: 10.1186/s13018-021-02337-1, PMID: 33691731 PMC7945340

[19] Liu J Liu Z Luo J Gong L Cui Y Song Q. Influence of vertebral bone mineral density on total dispersion volume of bone cement in vertebroplasty. Medicine (Baltimore). (2019) 98:e14941. doi: 10.1097/MD.0000000000014941, PMID: 30896660 PMC6709149

[20] Liu S Li H Wang D Qi X. Low bone mineral density promotes cement leakage in vertebra with compression fracture after percutaneous Vertebroplasty. J Biomaterials and Tissue Eng. (2017) 7:1355–9. doi: 10.1166/jbt.2017.1704

[21] Kim YW Kim JH Yoon SH Lee JH Lee CH Shin CS. Vertebral bone attenuation on low-dose chest CT: quantitative volumetric analysis for bone fragility assessment. Osteoporos Int. (2017) 28:329–38. doi: 10.1007/s00198-016-3724-2, PMID: 27480628

[22] Schreiber JJ Anderson PA Rosas HG Buchholz AL Au AG. Hounsfield units for assessing bone mineral density and strength: a tool for osteoporosis management. J Bone Joint Surg. (2011) 93:1057–63. doi: 10.2106/JBJS.J.00160, PMID: 21655899

[23] Lee S Chung CK Oh SH Park SB. Correlation between bone mineral density measured by dual-energy X-ray absorptiometry and Hounsfield units measured by diagnostic CT in lumbar spine. J Korean Neurosurg Soc. (2013) 54:384–9. doi: 10.3340/jkns.2013.54.5.384, PMID: 24379944 PMC3873350

[24] Amin MFM Zakaria WMW Yahya N. Correlation between Hounsfield unit derived from head, thorax, abdomen, spine and pelvis CT and t-scores from DXA. Skelet Radiol. (2021) 50:2525–35. doi: 10.1007/s00256-021-03801-z, PMID: 34021364

[25] Khoo BCC Brown K Cann C Zhu K Henzell S Low V. Comparison of QCT-derived and DXA-derived areal bone mineral density and T scores. Osteoporos Int. (2009) 20:1539–45. doi: 10.1007/s00198-008-0820-y, PMID: 19107384

[26] Fairbank JC Pynsent PB. The Oswestry disability index. Spine. (2000) 25:2940–53; discussion 2952. doi: 10.1097/00007632-200011150-00017 11074683

[27] Chiarotto A Maxwell LJ Ostelo RW Boers M Tugwell P Terwee CB. Measurement properties of visual analogue scale, numeric rating scale, and pain severity subscale of the brief pain inventory in patients with Low Back pain: a systematic review. J Pain. (2019) 20:245–63. doi: 10.1016/j.jpain.2018.07.009, PMID: 30099210

[28] Genant HK Wu CY van Kuijk C Nevitt MC. Vertebral fracture assessment using a semiquantitative technique. J Bone Miner Res. (1993) 8:1137–48. doi: 10.1002/jbmr.5650080915 8237484

[29] Lim J Choi SW Youm JY Kwon HJ Kim SH Koh HS. Posttraumatic delayed vertebral collapse: Kummell’s disease. J Korean Neurosurg Soc. (2018) 61:1–9. doi: 10.3340/jkns.2017.0505.010, PMID: 29354230 PMC5769843

[30] Nieuwenhuijse MJ Bollen L van Erkel AR Dijkstra PDS. Optimal Intravertebral cement volume in percutaneous Vertebroplasty for painful osteoporotic vertebral compression fractures. Spine. (2012) 37:1747–55. doi: 10.1097/BRS.0b013e318254871c, PMID: 22433500

[31] Lee KA Hong S Lee S Cha I Kim B Kang E. Analysis of adjacent fracture after percutaneous vertebroplasty: does intradiscal cement leakage really increase the risk of adjacent vertebral fracture? Skelet Radiol. (2011) 40:1537–42. doi: 10.1007/s00256-011-1139-x, PMID: 21399934

[32] Ding J Zhang Q Zhu J Tao W Wu Q Chen L. Risk factors for predicting cement leakage following percutaneous vertebroplasty for osteoporotic vertebral compression fractures. Eur Spine J. (2016) 25:3411–7. doi: 10.1007/s00586-015-3923-0, PMID: 25850391

[33] Kim YJ Lee JW Park KW Yeom JS Jeong HS Park JM. Pulmonary cement embolism after percutaneous vertebroplasty in osteoporotic vertebral compression fractures: incidence, characteristics, and risk factors. Radiology. (2009) 251:250–9. doi: 10.1148/radiol.2511080854 19332856

[34] Tomé-Bermejo F Piñera AR Duran-Álvarez C Román BLS Mahillo I Alvarez L. Identification of risk factors for the occurrence of cement leakage during percutaneous Vertebroplasty for painful osteoporotic or malignant vertebral fracture. Spine. (2014) 39:E693–700. doi: 10.1097/BRS.0000000000000294, PMID: 24583722

[35] Zhang H Xu C Zhang T Gao Z Zhang T. Does percutaneous Vertebroplasty or balloon Kyphoplasty for osteoporotic vertebral compression fractures increase the incidence of new vertebral fractures? A Meta-Analysis Pain Physician. (2017) 20:E13–28. doi: 10.36076/ppj.2017.1.E13, PMID: 28072794

